# Dislocation of the Symphysis Pubis during Pregnancy and Parturition

**Published:** 1867-03

**Authors:** D. C. O’Keefe

**Affiliations:** Prof. of Principles and Practice of Medicine, Atlanta Medical College


ATLANTA

Medical and Surgical Journal.

NEW SERIES.

Vol. VIII. MARCH, 1867. No. 1.

### ORIGINAL COMMUNICATIONS.

### ARTICLE I.

*Dislocation of the Symphysis Pubis during Pregnancy and Parturition.* By D. C. O'Keefe, M. D., Prof. of Principles and Practice of Medicine, Atlanta Medical College.

The occurance of this difficulty, in connection with and dependent upon utero-gestation, is more frequent than would be imagined without an investigation of the subject. We do not claim with Ambrose Pare, Sigault, Chaussiere, and others, that separation of the pubic bones at their symphysis, occurs as a normal process during labour, thereby increasing the antero-posterior diameter of the brim of the pelvis; but that a certain amount of softening and relaxation of the structures of the pelvic joints, producing more or less difficulty of locomotion and other movements of the body, takes place as a physiological result of the new and peculiar action set up in the system of the human female by pregnancy, seems to be well established. Distinguished authorities, a few centuries ago, maintained that a separation of the ossa pubis spontaneously occured in difficult labors, and this induced Sigault, while yet a medical student, in 1768, to conceive the idea, and apply it practically, of substituting section of the symphysis for the formidable Cæsarian operation.

This procedure excited great enthusiasm at the time of its promulgation, and secured the sanction of the Faculty of Medicine of Paris; a medal and pension were awarded to M. Sigault, based upon partial success in one case, and his patient was also granted a pension. Symphyseotomy, however, has no advocates at the present day; and M. Sigault and his patient, so far as we know, are the only beneficiaries of the operation.

The idea that dislocation of the symphysis pubis sometimes occured during parturition in the human female was based, no doubt, upon the well known fact in natural history, that such an event always takes place in some of the inferior animals. Some quadrupeds cannot long maintain the standing position during the last days of utero-gestation, in consequence of a relaxation of the joints of the pelvis, and in the guinea-pig, the symphysis pubis, at the time of labor, is widely separated to admit of the passage of the young.

That this relaxation and softening of the tissues of the pelvic articulations in the human female are events of rather frequent occurrence, any medical man may satisfy himself by investigating the condition of the pregnant women under his charge.

Many of them, to use their own quaint language, are "perfectly helpless;" walking on a level surface is performed well enough, but if the surface be rough and uneven, or if the effort be to ascend a flight of stairs, then the difficulty is very considerable.* The explanation of this condition is apparent: the pelvis is loosely connected with the vertebral column and the lower extremities with the pelvis in consequence of the softening of their ligaments and cartilages.

The symphysis pubis belongs to the class of joints called amphiarthrodial, which admit of a limited degree of gliding or swinging motion, and is formed of four ligaments and an interarticular fibro-cartilage, consisting of two oval shaped

---

* A lad under our care at the present time, who is otherwise well and comfortable, cannot get into bed, or change the position of her body in bed, without assistance.

plates, one covering the articular surface of each pubic bone.

This fibro-cartilage unites the two surfaces of the pubic bones in the same manner as the inter vertebral substance connects the bodies of the vertebræ, and a synovial membrane is sometimes found in the upper and posterior half of the articulation. This articulation sometimes becomes moveabel towards the latter term of pregnancy, and admits of a slight degree of separation of its surfaces. (Wilson's Anatomy, page 150.)

"By some admirable contrivance, the mobility of the intrinsic articulations of the pelvis is considerably increased during the latter periods of pregnancy, so that the coccyx may be pressed backward, causing an increase of five or six lines in the antero-posterior diameter of the outlet; while the symphysis pubis becomes susceptible of a slight separation, which increases (in a very slight degree, it is true, but sufficiently to merit notice) the dimensions of the brim of this cavity. This mobility, which is especially remarkable in a narrow pelvis, favors the process of labor in a singular degree." (Cruveilhier's Anatomy, page 159.)

"In progression, the whole pelvis receives the concussions, when they proceed from above or from below. Hence the ossa pubis are united by an intervening elastic fibro-cartilage; and any disturbance of this joint during pregnancy, or in the act of parturition, occasions great difficulty to the patient in walking, or even in maintaining the erect posture." (Todd & Bowman's Physiological Anatomy, page 140.)

It is thus seen that softening of the structures of the pelvic joints results, probably as a rule, from the pregnant condition in woman, according to our best anatomical and physiological authorities, and that this process of softening may exist to such an extent as to result in separation of the pubic bones at the symphysis, and sometimes, though rarely, in dislocation of the sacroiliac articulation. The following cases are adduced as examples of dislocation at the symphysis pubis: I was consulted about two months ago by a gentleman in regard to the condition of his wife from whom the following history of the case was elicited: She was 30 years old, of the lymphatic temperament—married at 19, and was now in the seventh or eighth month of her sixth pregnancy.

After the fifth month, a sense of soreness, weakness, and pain was felt in the region of the symphysis pubis, accompanied by difficulty in walking, especially upon a rough or uneven surface, or in ascending stair steps, or turning the body in bed. These difficulties increased with the advance of pregnancy, and a grating sound could be heard from the region of the pubis, not only by the patient, but by any one in her vicinity, whenever such movements of the body or lower extremities were made as would tend to move the pubic bones on each other at the symphysis. This displacement of the bones was attended with considerable pain in the pubic region and a sense of weakness approaching to syncope. Toward the termination of the term of utero-gestation, the function of locomotion was performed with so much pain and difficulty, that the patient was confined, most of the time, to the sitting or recumbent position. This difficulty in walking under such circumstances is easily understood. When one foot is raised, the weight of the whole body rests for the time being on the other, which, through the head of the femur in the acetabulum, presses up the corresponding side of the pelvis, and in this way produces the displacement, friction, and grating at the symphysis. This difficulty existed in the last three of her five former pregnancies, commencing three months before their termination, and although her labors were natural and easy, her convalescence was slow and troublesome.

Rest in the recumbent posture for two months after delivery seemed to restore the integrity of the joint, and she suffered no more inconvenience from it. Her labor in the present instance was marked by nothing unusual, and terminated to my entire satisfaction. She is now (February, 1867) passing through her convalescence with rather more comfort than usual, in consequence of a broad stout bandage applied round the hips. I attempted to examine the symphysis during the progress of the labor, with a view, if possible, to detect the dislocation, but the manipulation was attended with so much pain that nothing satisfactory was determined.

The lady and her husband are intelligent and reliable people, and I have, therefore, no doubt whatever of the correctness of their impressions and statements as to the facst of this case.

Dr. R. J. Massey, of the Drughouse of Massey, Swanson & Co., of this city, has kindly placed at my disposal the following history of cases that have come under his observation. I ought, perhaps, to state that the subject of his first case is the sister of the lady whose case has just been given, and that he considers the pubic joint as still imperfect, in which opinion, from my knowledge of the case, I fully concur.

"Mrs. ——, aged 19, somewhat strumous in her habit, was confined with her first child, on the 15th of July, 1852. Parturition was natural, but exceedingly protracted, even for a primipara. The passage of the head through the lower strait lasted eight or ten hours. The child was large, and weighed 10½ lbs. On the next day a partial separation of the ossa pubis at the symphysis was discovered by changing her from the dorsal posture to the left side. This was plainly perceptible to the touch, but no distinct crepitation could be heard. Rest and recumbent posture soon achieved partial restoration. At the next confinement, eighteen months afterwards, there was no separation. The child, however, at this time, was small, weighing only seven and a half pounds.

At the next confinement, four years afterwards, there was the same condition of affairs as at the first. Rest in the recumbent posture for two months restored the symphysis to partial integrity, and an exemption from further childbearing prevents the lady from suffering much inconvenience or pain, except a sensation of weakness and fatigue when ascending or descending stairs, or stepping over high places.

In August, 1856, near Penfield, Ga., I attended a negro woman, aged 34, in her fourth confinement. After an easy and rapid parturition, I detected, upon turning her from the dorsal position, a partial dislocation, attended with no distinct crepitation to the ear, but a sensation of grating to the touch. Recumbent posture and rest sufficed to bring about a partial restoration after a few months. After that I lost sight of the case. Feeling unusual concern in this interesting, though unusual abnormal condition, I consulted that eminent physician, and our mutual friend, the late Dr. W. S. Meire, of Madison, Ga., who informed me that he met a similar case in a negro woman, who suffered but little inconvenience, save restricted locomotion a few months before and after confinement. Recovery each time was partial, but at the next confinement there would be a repetition of this abnormal condition. When last heard from, she was still having children, about every eighteen months."

Dr. J. M. Johnson, of this city has been good enough to furnish me the notes of a case that occurred to him as follows:

"Mrs. H., a lady of refinement, education, and social position, aged 31 years, the mother of five children; subject to attacks of rheumatism in the knee and shoulder joints, large and fleshy—weighing 160 lbs.—has always had very easy deliveries until the last, which was more protracted and painful. She felt pain and straining in the symphysis pubis, attended with soreness in the middle of the pelvis, and weakness in her hips, which made it impossible to turn when in bed, and even yet, sixteen months since the trouble began, is obliged to roll out of the bed, and get in again to make the change. For several months she walked with difficulty, and yet does; and only attempts it for short distances. In attempting to lift a burden with one hand, she not only feels and hears the crepitation, but also feels the whole frame-work of the pelvis give way. I am satisfied that her opinion is correct, and that there is permanent separation of the pubic bones at the symphysis.

Otherwise than as stated, her health is good."

It remains but to add the opinions of surgical and obstetrical writers on the subject. "A separation of this joint (the symphysis pubis) occasionally occurs during utero-gestation, in consequence of softening of its fibro-cartilage, allowing the two bones to ride slightly upon each other. A case of this kind was under my observation not very long ago. The woman was in her fifth pregnancy, and the dislocation, beginning about a month before her confinement, was so great that she could not walk, or turn in bed without extreme distress. The parts were exquisitely tender, on pressure, and upwards of five weeks elapsed after parturition before they regained their healthy condition. Rest, recumbency, and leeches constitute the proper treatment aided, where the patient is able to move about, by a belt with a pad on the pubes." (Gross's Surgery, vol. 1, p. 1089.)

"With the exception of the caccyx, the movements of the bones of the pelvis, upon each other, in the unimpregnated state, are extremely limited. Some authorities have maintained that during labor all the pelvic symphyses are relaxed to a slight extent, while others have believed that the joints remain entirely unaltered at this time. This supposition led to the now abandoned operation of Sigault, for the division of the symphysis pubis in cases of contracted pelvis. Some have even believed that during gestation osseous matter is absorbed from the pelvis and carried into the circulation to supply the wants of the fœtus. Others contend that no change whatever occurs in the bones or joints of the pelvis in the course of parturition. We know, however, as Denman pointed out, that in rare cases, the sacro-iliac and pubic articulations become so relaxed as to cripple the subjects of this affection for a considerable time after labor. It is also known, from dissections of women dying during or immediately after labor, that the cartilages of the joints of the pelvis are found to be softer and more vascular than usual at this time. * * * * * * *

These facts afford good reasons for believing that in many cases a slight amount of relaxation of the pelvic articulations does take place in the human female during delivery." (Tyler Smith's lectures on obstetrics, p. 292.)

Churchill believes that it does not take place as a natural process, but that it occurs occasionally as an accident.

Burns says that, by some morbid affection of the symphysis, it may yield and become loosened during pregnancy, or may be separated during labor. He gives the symptoms and treatment of the dislocation, and the mode of detecting it. "If the bones be fully disjointed, then, by placing the finger on the inside of the symphysis, and the thumb on the outside, we can readily perceive a jarring, or motion, on raising the thigh."

Chailly, in his work on midwifery, treats the subject so much to the point that, at the risk of being tedious, I will copy his remarks in full: "The softening of the ligaments which unite the bones of the pelvis is of constant occurrence during pregnancy; but we should regard it rather as the accomplishment of a general law, which governs all beings, than as a necessary consequence of parturition in the female. If this softening of the symphysis is indispensable to the completion of the generative functions in certain animals, in which the pelvis is very narrow, it is not so in the human species.

In the various pelves of women recently delivered which have been submitted to my observation, I have never found this relaxation sufficiently distinct to increase the diameters of the pelvis a few millimetres, unless the woman had, during her gestation, experienced all the inconveniences which characterize a considerable separation of the bones, and which is not a physiological condition, but constitutes actual disease.

In this case, walking and standing are attended with great difficulty, fatigue, and pain. During labor, the auxiliary muscles of the uterus, finding no fixed point of support in the pelvis, act painfully on the symphysis, and afford no aid to the uterus in its contractions.

The malady, the causes of which are little known, and which some authors have attributed to rachitis, commences by dull pains in the articulations of the pelvis, loins, hips and thighs: motion becomes slow, difficult, and so painful as to render progression almost impossible. Sometimes the the pains are so acute that the patient, even while in bed, cannot make the slightest movement without experiencing them. When she rises and walks, she feels a sensation of great weakness and vacillation; it seems as if her body was about to slip between her thighs, and the pelvis about to separate. If the disease progresses in consequence of successive pregnancies, the disjunction, separation, and relation of the symphysis become so marked that, on moving the inferior extremities, the mobility of the symphysis is not only ascertained, but there is heard a cracking noise. Then it becomes utterly impossible for the woman to move her lower limbs: she is obliged to be raised by assistants; and the sensibility and swelling of the parts are such that the slightest touch causes pain. The consequence of this condition manifest themselves particularly after delivery, and are not accompanied by positive danger unless it proceeds from an organic vice." Here follows the treatment which is not necessary to detail.

The subject will be concluded with the interesting remarks of Prof. Meigs, from his treatise on obstetrics.

"Many people among the mass of society, suppose that in every labor the joints become relaxed, in order to let the child pass through the bones: and a good many ladies take a spoonful of oil of olives or Palma Christi, with a view to promote this desirable relaxation as they esteem it to be.

"I have known a young thing to take the trouble, nightly, to anoint the mons veneris for a long period, with lily ointment, to soften the joint.

"It is understood, however, by the anatomist, that these joints do not become open and relaxed as a normal effect of gestation, of labor, or of endermic or therapeutical measures, resorted to for that end. Yet they do, in some persons, relax, to their great injury or inconvenience. As to the symphysis pubis, I have on many occasions found it to be quite loosend, and admitting of motion. One of my patients, whom I have succord in many of her cofinements, has greatly suffered from relaxation of the symphysis pubis during the several last weeks of her pregnancies. The articulation becomes so loose as to make a very considerable cracking sound, when she would turn in bed, or walk; and she has been good enough, in order that I might verify the fact, to allow me to cause the motion by pressing with my hands on the opposite spinous processes of the iliac bones, by which means I could cause the two opposite pubes to approach or separate from each other, or ride up and down, passing each other in the direction of the length of the symphysis.

"When the patient, in such a state of the interpubal ligament, stands on the right foot, the right pubis rises upwards, while the left descends, and vice versa, so that the act of walking is not only attended with pain, but with tottering and uncertainty.

"The lady in question gives birth to children weighing ten and twelve pounds, but she has commonly recovered from the relaxation within about forty days after the birth of the child, and her pubic joint remains perfectly strong and efficient, until in an advanced stage of the next gestation, the pressure and infiltration come to loosen and dispart the bones again. This lady has been fourteen times pregnant, and has given birth to twelve children at term. The joint did not give way until the sixth accouchement, which occurred October 20th, 1833.

"The child weighed upwards of twelve pounds. The motion of the symphysis was very obvious, and very painful. She recovered from it, however, and did not feel it again until near the close of pregnancy, which was concluded on the twelvth of December, 1835, by the birth of a son. A daughter was born October 30th, 1837, which produced the relaxation.

"She soon got over this, and in the next pregnancy and confinement felt nothing of it. This labor was on the second of September 1843. When the child was three months old the relaxation took place, and was long troublesome. She was again pregnant in 1845, but had no return of the inconvenience in the gestation or lying-in, which occured January 20th, 1846. The joint gave way again soon after her last accouchement, August 17th, 1848; she discovered it on the 20th day of the month, and it is so moveable, that a cracking sound is produced by turning in bed."

